# A 13-Year-Old Girl Affected by Melanocytic Tumors of the Central Nervous System—The Case

**DOI:** 10.3390/ijms25179628

**Published:** 2024-09-05

**Authors:** Emilia Nowosławska, Magdalena Zakrzewska, Beata Sikorska, Jakub Zakrzewski, Bartosz Polis

**Affiliations:** 1 Department of Neurosurgery, Institute of Polish Mother’s Health Centre in Lodz, Rzgowska 281/289, 93-338 Lodz, Poland; bartosz.polis@iczmp.edu.pl; 2 Department of Molecular Pathology and Neuropathology, Medical University of Lodz, Pomorska 251, 92-213 Lodz, Poland; magdalena.zakrzewska@umed.lodz.pl (M.Z.); beata.sikorska@umed.lodz.pl (B.S.); 3 Faculty of Medicine, Medical University of Lodz, Kosciuszki 4, 90-419 Lodz, Poland; jakub.zakrzewski@student.umed.lodz.pl

**Keywords:** central nervous system melanoma, molecular analysis, neurocutaneous melanocytosis, pediatric melanoma, neurocutaneous phakomatosis

## Abstract

Primary intracranial melanoma is a very rare brain tumor, especially when accompanied by benign intramedullary melanocytoma. Distinguishing between a primary central nervous system (CNS) lesion and metastatic melanoma is extremely difficult, especially when the primary cutaneous lesion is not visible. Here we report a 13-year-old girl admitted to the Neurosurgery Department of the Institute of Polish Mother’s Health Centre in Lodz due to upper limb paresis. An intramedullary tumor of the cervical C3–C4 and an accompanying syringomyelic cavity C1–C7 were revealed. The child underwent partial removal of the tumor due to the risk of damage to spinal cord motor centers. The removed part of the tumor was diagnosed as melanocytoma. Eight months later, a neurological examination revealed paresis of the right sixth cranial nerve, accompanied by bilateral optic disc edema. Diagnostic imaging revealed a brain tumor. The girl underwent resection of both detected the tumors and an additional satellite lesion revealed during the surgery. The removed tumors were diagnosed as malignant melanomas in pathomorphological examination. Molecular analysis revealed *NRASQ61K* mutation in both the intracranial and the intramedullary tumor. It should be noted that in cases where available evidence is inconclusive, an integrative diagnostic process is essential to reach a definitive diagnosis.

## 1. Introduction

Primary intracranial melanoma is a very rare brain tumor, especially when accompanied by benign intramedullary melanocytoma [1,2,3]. Central nervous system (CNS) melanocytomas are derived from epithelial melanocytes [3]. It is generally difficult to distinguish primary CNS melanoma from metastatic primary skin melanoma [4,5,6,7,8,9,10]. Children affected by congenital skin birthmarks reveal more common primary CNS melanomas than primary skin melanomas [6,7,10]. In most cases, primary CNS melanoma is accompanied by neurocutaneous melanosis [1,2]. This rare phacomatosis may not be easily noticeable, especially when there is no large mole (more than 20 cm in diameter in adults and 6–9 cm in infants), but small melanotic patches (more than three) [1,2]. The presented case is particularly interesting because it is an example of the coexistence of primary intracranial melanoma with primary intramedullary melanocytoma. All initial neurological symptoms were characteristic of the compression of neuronal structures by a benign tumor. It is difficult to answer the question of what the etiology of the intracranial frontal lobe melanoma was. Was it primary malignant transformation of intracranial melanocytes in situ, regardless of the primary intramedullary lesion? Did the intracranial melanoma arise from malignant transformation of melanocytes which derived from the intramedullary melanocytoma and then spread to the intracranial space? This seems possible, especially if malignant transformation of leptomeningeal melanocytes has occurred, as in the published case of melanoma regrowth after melanocytoma removal [3,6]. Due to the atypical clinical picture of both independent tumors (intracranial and intramedullary), the presented case may have potential educational value. The case study should be helpful in determining the appropriate strategy for the early detection of intracranial melanoma.

## 2. Case Presentation

**Figure 1:**
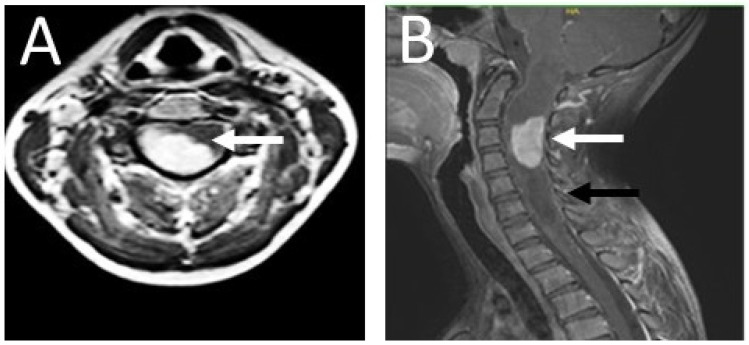
Contrast-enhanced T1 MRI of the patient’s cervical spine. (**A**) The axial section at the C2 level; (**B**) the sagittal section. The photos were taken before the procedure. An intramedullary tumor is located in the spinal canal between the C2–C4 levels (white arrow), accompanied by a syringomyelic cavity extending throughout the spinal cord from the hindbrain to the cervical spine at the C7 level (black arrow). Due to the dynamic neurological symptoms, characteristic of the expansive process in the spinal cord, and the need for urgent surgery, imaging diagnostics were limited to the spinal cord and the cervicocranial border. An MRI of the brain was performed at a later stage.

On 27 April 2018, a 13-year-old girl was admitted to the Department of Neurosurgery at the Polish Mother’s Memorial Hospital, Research Institute. The patient complained of progressive weakness of the right upper limb, accompanied by numbness of the first, second, and third fingers of the right hand. Sensory disturbances began in November 2017. On admission, neurological examination revealed weakness of the muscles of the right hand (Lovett 3) and right forearm (Lovett 4). She showed lack of postural control and a right-sided Romberg’s sign. The right upper limb paresis with weak tendon reflexes was caused by a lower motor neuron lesion. Magnetic resonance imaging (MRI) revealed a right-sided C3–C4 intramedullary tumor. It was accompanied by a syringomyelic cavity C1–C7 (Figure 1). The fluid reserve surrounding the cervical spine at the level of the lesion was completely depleted.

**Figure 2:**
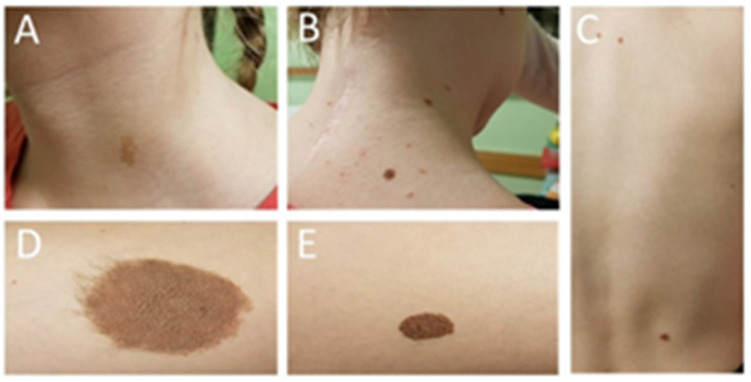
Photos of the patient’s skin pigment spots: (**A**) a skin mole on the neck removed after dermatoscopy (melanoma was excluded at the time); (**B**) the remaining part of the skin moles on the neck; (**C**) birthmarks on the skin of the back; (**D**) a hairy birthmark on the left thigh; (**E**) a nodule birthmark on the skin of the right thigh removed after dermatoscopy (melanoma was excluded at the time).

The patient’s skin was covered with several pigment spots (Figure 2). The most visible were those located on the left thigh (3 cm pigmented hair patch) and on the right thigh (2 cm lumpy pigmented birthmark). The rest of the birthmarks were found on the neck and back skin.

**Figure 3:**
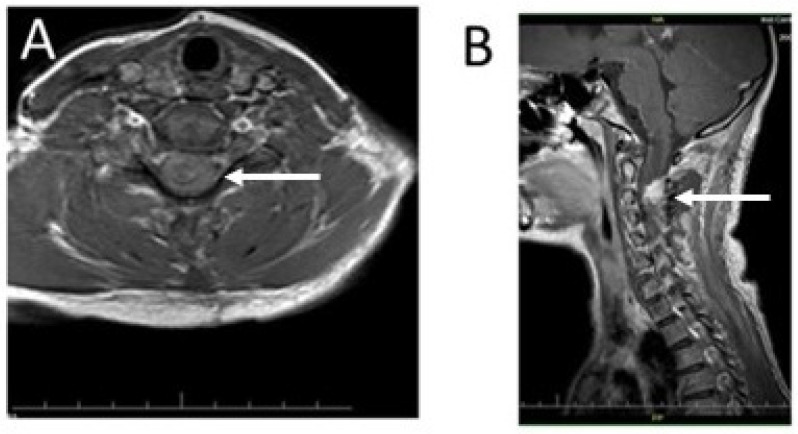
Imaging diagnostics of the patient’s cervical spine using T1-weighted MRI with contrast enhancement 48 h after the procedure: (**A**) an axial section of the cervical spine at the C2 level with clearly visible reduction of the syringomyelic cavity (arrow); (**B**) a sagittal scan of the cervical spine; the remaining part of the tumor mass in the cervical spine is visible as a hyperintense focus in contrast to the image of the spinal cord (arrow).

Due to the observed neurological deficits, the girl was qualified for urgent surgery. The tumor removal was performed by C2–C4 laminectomy. The motor-evoked potential (MEP) and the somatosensory-evoked potential (SEP) were monitored throughout the procedure. Due to the evoked potential signal disturbance, the innermost layer of the pathological lesion, tightly connected with the cervical spine, was left intact. Although the evoked potential signal returned to its initial level, the girl’s neurological condition worsened after the operation. The patient presented right-sided hemiparesis. The girl showed equal muscle weakness in the upper and lower limbs (Lovett 3). An MRI of the cervical spine, carried out 48 h after the surgery, confirmed the presence of some tumor remnants. The syringomyelic cavity was fully decompressed (Figure 3).

**Figure 4:**
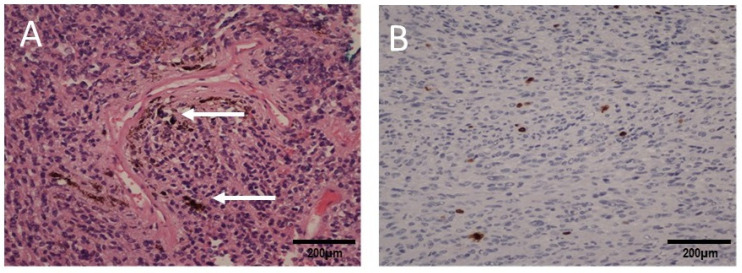
Microscopic images of the cervical spinal cord tumor: (**A**) H&E staining shows oval and spindled cells containing pigment (white arrows), magnification 200×; (**B**) Ki67 immunohistochemical staining showing a low proliferation index of the tumor cells (<5% of positive cells), magnification 200×.

The patient’s neurological condition improved a week later. The right-sided hemiparesis partially withdrew. Muscle strength improved to four degrees on the Lovett scale (Lovett 4). The girl was able to walk. Based on the histopathological examination, the final diagnosis of the removed tumor was established as melanocytoma (Figure 4).

**Figure 5:**
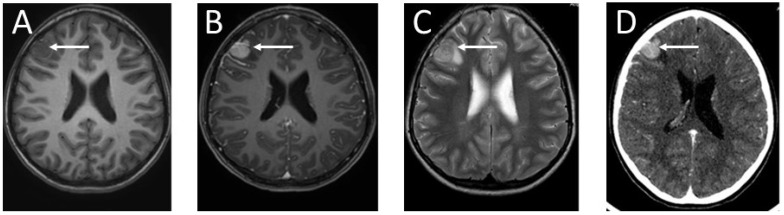
Imaging of the brain tumor: (**A**) T1-weighted MRI of the patient’s head without contrast enhancement—the brain tumor is visible as a hypointense lesion in contrast to the white matter, located in the right frontal lobe; (**B**) contrast-enhanced T1-weighted MRI—the mass in the right frontal region shows intense contrast enhancement; (**C**) T2-weighted head MRI—the tumor in the right frontal area is visible as a hyperintense lesion in contrast to the white matter; (**D**) CT diagnostic imaging after contrast enhancement—the tumor in the right frontal region is visible as a hyperdense lesion in contrast to the white matter of the brain. The arrows indicate the tumor location.

The girl was discharged from hospital. She was under the care of the outpatient clinic. Eight months later, the patient was admitted to hospital due to an epileptic seizure. The child’s mother claimed that the girl had complained of double vision a month earlier. Neurological examination revealed paresis of the right sixth cranial nerve. The child complained of diplopia when looking straight ahead and to the right. A fundoscopy revealed bilateral optic disc edema. The girl underwent a two-phase contrast-enhanced computed tomography (CT) followed by an MRI (Figure 5). In the right frontal area, a well-enhanced, spherical tumor, measuring 16 × 17 × 17 mm, was visible. It was surrounded by a 7 mm peritumoral edema zone. The ventricular system of the brain was slightly enlarged, but without signs of active hydrocephalus.

**Figure 6:**
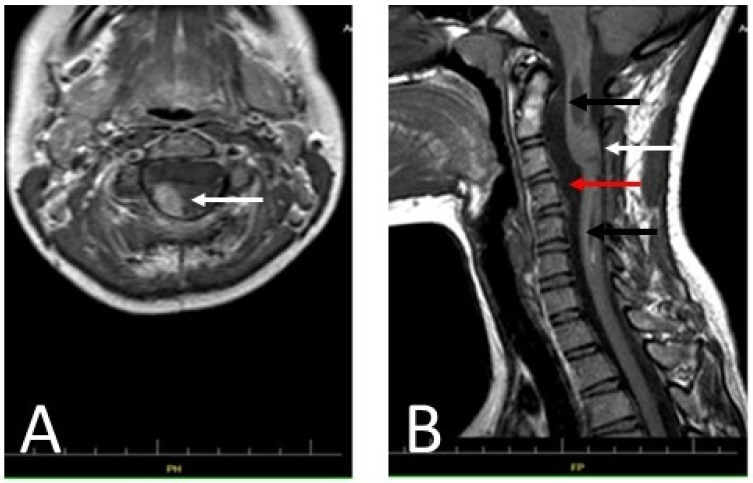
Diagnostic T1-weighted MRI images of the patient’s cervical spine: (**A**) An axial section at the C2 level of the patient’s cervical spine made after administration of a contrast agent. The remainder of the tumor showed no apparent progression compared to the MRI performed 48 h after the first treatment (arrow). (**B**) A sagittal section of the patient’s cervical spine (without contrast). The photo shows decompression of the syringomyelic cavity (black arrows) and the remains of the partially removed tumor, visible as an isointense lesion in the spine (white arrow). The reserve of cerebrospinal fluid in the spinal canal is clearly increased (red arrow). The photo shows no signs of tumor progression.

Additionally, an MRI of the cervical spine was performed (Figure 6). The remaining part of the cervical spine tumor, not resected during the previous surgery, showed no changes compared to the former MRI examination.

**Figure 7:**
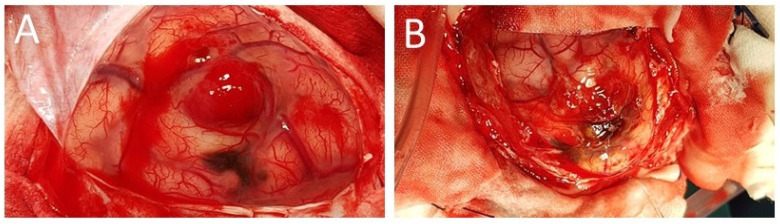
An intraoperative view of the surgical field. The right frontal lobe of the brain is exposed through a right frontal craniotomy: (**A**) fresh blood in the tumor mass located above the subarachnoid black satellite lesion; (**B**) the main focus of the tumor after removal of the blood clot, visible above the dark subarachnoid lesion.

The girl was operated on. A right frontal craniotomy was performed. Previous spontaneous bleeding into the tumor was detected intraoperatively (Figure 7). An additional satellite pathological lesion was found, initially invisible in MRI. The pathological black lesion in the subarachnoid space resembled the larger one. Both tumors were completely removed during the surgery. The postoperative course was complicated by bleeding in the surgical area, requiring additional surgery to remove the intracerebral hematoma. Antiepileptic treatment of levetiracetam was administered.

**Figure 8:**
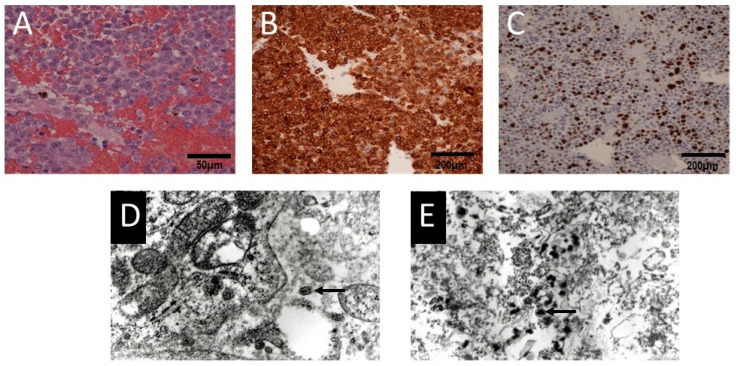
Microscopic images of the intracranial tumor: (**A**) H&E staining shows pleomorphic epithelioid cells, magnification 400×; (**B**) immunoexpression of MelanA in the tumor cells, magnification 200×; (**C**) Ki67 immunohistochemical staining showing a high proliferation index of the tumor cells (>30% of positive cells), magnification 200×; (**D**,**E**) transmission electron microscopy images of the melanotic tumor cells (arrows), magnification 25,000×.

A malignant melanoma was diagnosed on the basis of pathomorphological examination of the samples taken from both intracranial tumors, which demonstrated pleomorphic cells with MelanA immunoreactivity and an elevated proliferative index (Figure 8).

**Figure 9:**
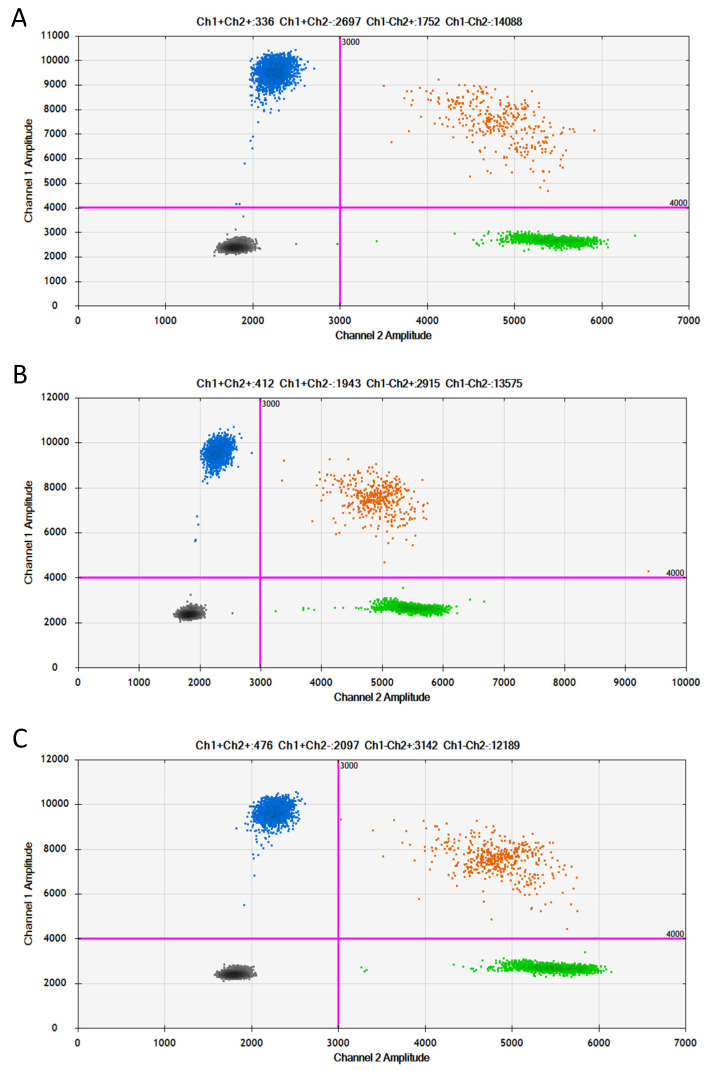
Two-dimensional plots of ddPCR analysis of *NRASQ61K* mutation: (**A**) intramedullary tumor sample, ratio = 1.49; (**B**) brain tumor sample, ratio = 0.688; (**C**) fascial sample, ratio = 0.687. Purple lines indicate thresholds for distinguishing positive (blue cluster), positive/wild-type (orange cluster), negative (gray cluster), and wild-type (green cluster) signals. The mutation frequency of genes was quantified by the ratio of mutant droplets (only HEX positive) to wild-type droplets (HEX/FAM double positive).

Melanocytic tumors are characterized by various mutations in MAPK (mitogen-activated protein kinase) pathway genes, of which *BRAF* alterations are the most common [11]. Because of this, the patient’s samples were tested for somatic hotspot mutations in *BRAFV600E* and *NRASQ61K* using the extremely sensitive QX100 Droplet Digital PCR System (BioRad, Hercules, CA, USA). The analyses were performed with *BRAF V600E* (FAM-labeled mutant assay: dHsaCP2000027, and HEX-labeled wild-type assay: dHsaCP2000028) and *NRASQ61K* (FAM-labeled mutant assay: dHsaCP2000067, and HEX-labeled wild-type assay: dHsaCP2000068) primers and probes to confirm or exclude the presence of driver gene mutations. Each experiment included a negative no-template control and positive control (a sample with a double-stranded, synthetized, mutated DNA fragment (Integrated DNA Technologies (IDT), Redwood City, CA, USA)). After PCR amplification, the samples were analyzed with the QX200 Droplet Reader (Bio-Rad, Hercules, CA, USA) for fluorescent measurements of the FAM- and HEX-labeled probes (Quanta Soft software, version 1.7). The fractional abundance was based on the ratio between mutant and wild-type droplets after correction using the Poisson distribution. The molecular analysis excluded the *BRAF* mutation and revealed the presence of the *NRASQ61K* c.181C>, a mutation which was confirmed for each tumor and fascial sample (Figure 9).

**Figure 10:**
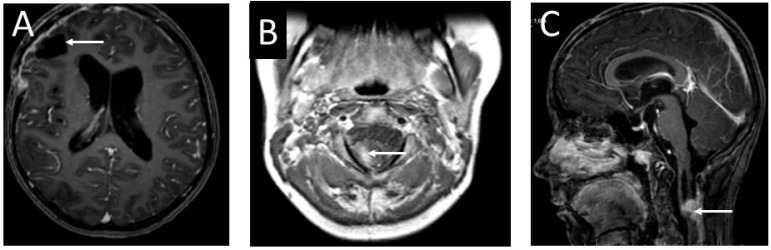
Contrast-enhanced T1 MRI images of the head and cervical spine of the 13-year-old girl before the valve implantation: (**A**) an axial section of the head. The photo does not show progression of the tumor (arrow). (**B**) MRI of the cervical spine—an axial section. Visible remnants of the tumor diagnosed as melanocytoma (arrow). (**C**) An MRI—sagittal section of the cervical spine. The residual tumor mass shows no enlargement compared to previous diagnostic images (arrow).

The patient was referred to an oncological center for further diagnosis and treatment. The child underwent dermatoscopy and two skin birthmarks were selected for removal (Figure 2). A histological examination did not show any malignant transformation in the analyzed samples. The child was qualified for stereo-radiotherapy. Three months later, the patient developed hydrocephalus and required shunt implantation. The biphasic MRI showed no metastases in the subarachnoid space and no progress of the residual cervical spine tumor (Figure 10). After discharge from hospital, the girl underwent chemotherapy.

Despite the oncological treatment, progressive clinical and neurological deterioration was observed. The child’s demise took place six months later due to diffuse infiltration of the CNS and insufficiency of the brain stem.

## 3. Discussion

Primary melanoma of the CNS is a very rare pathological lesion in the pediatric population, especially when accompanied by other benign, independent tumors such as melanocytomas; therefore, clinicians should always thoroughly analyze the clinical courses of such rare cases. According to the literature, fewer than 30 cases were described in the years 1989–2016 and primary CNS melanoma is thought to occur in as few as 1% of all melanoma cases [6,12,13,14,15,16]. It also accounts for 0.07% of all primary brain tumors [5,8]. In adulthood, primary CNS melanoma arises from leptomeningeal melanocytes, probably as a result of sporadic mutations in cancer-related tumor suppressor and DNA repair genes [14,17].

In the presented case, the clinical picture of the intracranial melanoma was dominated by double vision caused by increased intracranial pressure. Despite a lack of visible signs of intracranial metastases in MRIs and complete tumor resection, the increased intracranial pressure persisted. The main clinical symptoms of primary CNS melanoma in children are the same as in other brain lesions. The tumor mainly causes increased intracranial pressure, neurological deficits, subarachnoid hemorrhage, epileptic seizures, and cranial nerve dysfunction [6,14,18]. In children, the intracranial location of primary CNS melanoma is considered to be more frequent than its location in the spinal cord [16].

The main theory of origin of primary CNS melanoma in this age group is that the disease is caused by neural crest melanoblast aberrations that occur on the twenty-second day of embryogenesis [12,14,15,16,17,18,19]. Leptomeningeal melanoblasts may be another source of CNS melanoma in children [14,18,20]. Another theory explains the development of primary CNS melanoma by the mutation of mesoderm-derived pigment cells transported in blood vessels. The ectodermal theory states that CNS melanoma originates from abnormal embryonic epidermal cells [15]. But most important is that CNS melanoma in children is in most cases congenital and it is usually accompanied by neurocutaneous melanosis [13,18]. 

The main known risk factor for melanoma in the pediatric population is the presence of congenital melanocytic naevi (CMN) [6,21]. The incidence of primary CNS melanoma accompanied by CMN reaches up to 10–15% of all noted-to-date cases [6]. It is currently assumed that one-third of patients with CMN also suffer from all types of melanomas. Patients affected by CMN are extensively investigated for molecular abnormalities which could be used as prognostic factors and goals for targeted therapy, especially when the clinical course of such tumors is rather fulminant. The average age of death is 3 to 9 years [6]. Our patient, despite being older, had a fatal outcome, too. 

When primary CNS melanoma is part of neurocutaneous melanomatosis, its clinical picture is complemented by the clinical features of this phacomatosis [18,19,21]. Therefore, it is important to examine the patient’s skin to detect multiple small or a single large melanocytic nevus. It is also important to remember that primary intracranial CNS melanoma may be accompanied by benign spinal tumors such as CNS melanocytoma [13,15,18,22,23]. 

As mentioned above, the coexistence of this phacomatosis with primary CNS melanoma is characterized by a very fulminant course in children [6,15,23]. In the presented case, the patient was diagnosed with coexistence of intracranial melanoma with intraspinal melanocytoma. Moreover, our patient also had melanotic nevi skin birthmarks, but none of them showed malignant transformation. In the presented case, the criteria for the neurocutaneous melanomatosis were met due to the presence of at least three smaller birthmarks and a benign CNS melanocytic tumor [23]. During the whole observation period of the patient, the melanotic skin birthmarks did not show any neoplastic transformation (Figure 2). 

The tumor located in the cervical spine, although not completely removed, showed no signs of regrowth throughout the entire clinical period. Primary CNS melanoma shows some MRI similarities to other meningeal lesions and requires differentiation from melanocytic schwannoma, meningioma, melanocytoma, and melanin deposits in leptomeninges [20,22]. On MRI T1-weighted images, the contrast enhancement of the pathological lesion was clearly visible. In T2-weighted images, the pathological mass was hypointensive [14]. The similarity of MRI images of all the mentioned lesions requires the correct histopathological diagnosis established in the differentiation process. Therefore, the observation of the clinical course of this particular case leads to the conclusion that despite the absence of intracranial neurological symptoms, an MRI of the brain and spinal cord should have been performed as soon as possible. MRI screening is recommended for children with CMN who are less than 6 months of age. At this age, before full myelination, it is possible to visualize the melanin signal during the examination [6]. 

The intraoperative appearance of the tumor was typical for these melanotic lesions described in the literature [7,10,20]. CNS melanoma in children does not differ histologically from the same type of tumor in adults and is characterized by a variety of pleomorphic cells, numerous mitoses, and focal necrosis regardless of the location [2,13,18]. Malignant melanoma was diagnosed on the basis of pathomorphological examination of the samples taken from both tumors, which demonstrated pleomorphic cells with MelanA immunoreactivity and an elevated proliferative index (Figure 4 and Figure 8). Moreover, an investigation using the transmission electron microscope revealed the presence of melanotic tumor cells (Figure 8).

Taking into account the literature data, we also performed molecular analyses of the plausible causative genes of this patient. In children with melanocytic CNS lesions accompanied by melanotic congenital syndromes, the *NRAS* mutations have been demonstrated [6,21]. So far, a limited number of cases of primary CNS melanoma affected by the *NRASQ61K* mutation without coexistence of neurocutaneous melanomatosis has been described [6,18,21]. Here we show that *NRASQ61K* could be a part of the clinical picture of children with CNS tumors accompanied by a neurocutaneous melanotic syndrome (Figure 9). The presence of the mutations in non-affected tissue is indicative of a neurocutaneous syndrome, which is caused by a post-zygotic missense variant of *NRAS* [24]. NRAS is one of three isoforms of the RAS family of guanosine triphosphate (GTP) binding and hydrolyzing proteins, which are involved in cell growth by activating key signaling pathways, including MAPK. Substitution of Q61K results in a constitutively active NRAS protein and is therefore associated with tumor formation. Melanocyte-derived tumors are very often associated with a mutation in the hotspot genes involved in the mitogen-activated protein kinase (MAPK) pathway (RAS, RAF, MEK, ERK) [22]. A change in MAPK signaling leading to overexpression of p-ERK, activation of the GNA 11 pathway, and *NRAS* mutation has been found to be an alternative to the common mutation of *GNAQ*, which encodes the Gαq subunit of heterotrimeric G-proteins [14,18,22,25]. The *BRAF* mutation occurs in 60–87.5% of skin nevi, but *NRAS* mutations occur in only 20% of them. In up to 80% of cases of large congenital nevi, *NRAS* mutations are found [22,26]. Molecular tests were very helpful in establishing the correct diagnosis. NRAS mutations are considered characteristic of primary CNS melanoma associated with neurocutaneous melanocytosis [27].

In the presented case, despite aggressive surgery, chemotherapy, and radiotherapy, the outcome was fatal. To date, there is no effective treatment in clinical practice. Complete tumor resection is not possible due to its malignant nature and does not lead to full recovery [6,12,15,17,21,28]. Stereotactic radiosurgery can be used in localized lesions [13]. Despite an early discovery of the neoplasm, the prognosis is generally poor [8]. In 50% of cases, progression-free survival is maintained for six months after diagnosis [12,16]. The role of chemotherapy in survival is unclear [12,15]. To date, Trametinib has been found to be effective in prolonging postoperative survival, especially in patients affected by the *BRAFV600* mutation [2,6,16,26]. To sum up, prognosis for primary melanoma is associated with the degree of mitosis, leptomeningeal dissemination, extent of surgical excision, and the tumor location. Despite all possibilities, patients may die due to diffuse intracranial dissemination [19]. 

## 4. Conclusions

In the described case, the most probable tumor stage of the local melanoma seems to be atypical neurocutaneous melanocytosis with no large and numerous pigmented nevi on the skin. The malignant transformation of melanocytes in the residual lesion after removal of the melanocytic tumor of the cervical spine might occur, even if no evidence of spread in the intra-arachnoid space was found on MRI. Perhaps performing a two-phase magnetic resonance imaging of the head when a spinal cord tumor was detected would have allowed an earlier detection of the intracranial melanoma. However, due to the very fulminant clinical course of the intracranial melanoma, it initially might not have been visible on diagnostic MRI. Furthermore, the presence of the mutations which are functionally associated with cell growth and survival makes them potential targets for anti-cancer therapies in the future, especially in cases characterized by poor prognosis.

## Data Availability

The datasets used and analyzed are available from the corresponding author on reasonable request.
